# Supplementary material for: Predicting resistance to fluoroquinolones among patients with rifampicin-resistant tuberculosis using machine learning methods
Source: PLOS Digit Health. 2022 Jun 30;1(6):e0000059. doi: 10.1371/journal.pdig.0000059 (PMC9518704; doi:10.1371/journal.pdig.0000059)
Supplement: S1 Text — Table A. The estimated area under the receiver operating characteristic curve (AUC-ROC) calculated using 5-fold cross-validation, for predictive models developed by different machine learning algorithms and feature selection methods. Figure A. The estimated area under the ROC curves (AUC-ROC) of predictive models that did not account for the local prevalence of resistance to FLQs, identified by different feature selection methods. Figure B. The estimated area under the ROC curves (AUC-ROC) of predictive models that accounted for the local prevalence of resistance to FLQs, identified by different feature selection methods. Figure C. The frequency of features identified as important using recursive feature elimination algorithm and random forest classifier among 200 bootstrap iterations (see §S4 in S1 Text). Figure D. Evaluating the performance of the random forest model that accounts for the local prevalence of resistance to FLQs using features identified by recursive feature elimination for varying classification threshold. Figure E. The optimal choice of the classification threshold for varying values of the policymaker’s trade-off threshold and the optimism-corrected utility of the random forest model to determine whether FLQs should be included or replaced with DLM for a patient with RR-TB. Figure F. The estimates F1 and Matthews correlation coefficient (MCC) scores for varying classification threshold for our final model (neural network classifier and permutation importance algorithm). (PDF) [file pdig.0000059.s001.pdf]

S1 Text: Additional Details and Analyses

"Predicting resistance to fluoroquinolones among patients with rifampicin-resistant tuberculosis using machine learning methods"

S1 Data cleaning and preprocessing

S1.1 Determining susceptibility and resistance to RIF and FLQs

Susceptibility or resistance to rifampicin (RIF) was determined based on the results of Xpert MTB/RIF test, which could be obtained at the point-of-care.

Susceptibility or resistance to fluoroquinolones (FLQs) was determined based on the susceptibility or resistance to ofloxacin, levofloxacin, and/or moxifloxacin. The susceptibility or resistance to these three drugs were determined based on LJ- and MGIT- based drug susceptibility tests. If either or both tests detected resistance to one of three drugs, such as ofloxacin, we classified the patient's *M tuberculosis* strain as resistant to ofloxacin. If both tests were negative or if one was negative and the other was missing, we classified the patient's *M. tuberculosis* strain as susceptible. If none of these conditions are met, we coded the resistance status as "missing."

If resistance to at least one of the three drugs was determined, we classified the patient's *M. tuberculosis* strain as FLQ-resistant. If susceptibility to all these drugs was determined, we classified the patient's *M. tuberculosis* stain as susceptible to FLQs. If none of these conditions are satisfied, we coded the resistance status as "missing".

S1.2 Preprocessing

We coded entities with no or unrealistic values as "missing." For each feature, the percentage of entities with missing values are provided in Table 1. As the occurrence of these "missing" values may not be completely random, we kept all records with "missing" values in our analysis to allow the development of predictive models that can work with missing observations. We used one-hot encoding to incorporate nominal categorical features (e.g., occupation, education, TB type). We standardized the only continuous feature in our dataset (i.e., age). For the feature 'number of household contacts' we grouped entities with values ≥ 11 into a single stratum and treated these features as ordinal categorical predictors (i.e., replacing its values with 0, 1, 2, …). For feature 'number of household contacts 18 or younger', we grouped entities with values ≥ 8 into a single stratum. We extrapolated the missing entries for 'number of household contacts' and 'number of household contacts 18 or younger' by replacing them with the mean values of each column. For the feature 'residing in a district with low, median, or high prevalence of resistance to FLQs' we replaced low, medium, and high values with 0, 1, 2.

S2 Machine learning and feature selection algorithms

We used the scikit-learn package to train logistic regression, neural network, and random forest models [1]. We set the class weight of the logistic regression models to be "balanced". Neural network models were trained using

the lbfgs solver with tanh activation functions and one hidden layer containing as many nodes as the number of features + 2. For the random forest models, we set the number of trees to be 100 and the minimal number of samples required to be at a leaf node to be 5.

To identify features with important predictive power and to remove features that would diminish the model's accuracy, we used recursive feature elimination (RFE) [2], L_1 regularization (L1) [3], and permutation importance (PI) [4] as feature selection methods for different classifiers. We used RFE, L1 and PI for logistic regression models, RFE and PI for random forest models, and PI for neural network model [1]. For L1, we set the regularization parameter to 0.2 and for PI, we set number of times a feature is randomly shuffled to 10.

To determine the optimal number of features for each model, we checked how the optimism-corrected estimates of AUC-ROC (calculated using the algorithm described below in §S4) varied by the number of features included (Figure A and Figure B). We chose the smallest number of features after which the optimism-corrected estimate of AUC-ROC became stable. To characterize the importance of each feature, we recorded the number of times each feature was identified as important within the iterations of the bootstrap algorithm describe below.

S3 Performance criteria

To evaluate the performance of the predictive models developed here, besides AUC-ROC, we also estimated the model's sensitivity and specificity, and its impact on the proportion of individuals with RR-TB who receive appropriate regimen and who unnecessarily receive DLM. These performance criteria are defined below (we use $\mu_{FLQ\text{-}R}$ to denote the prevalence of resistance to FLQs among individuals with RR-TB):

Sensitivity ($\boldsymbol{\alpha}$):

$$\frac{true\ positive}{true\ positive + false\ negative}$$

Specificity ($\boldsymbol{\beta}$):

$$\frac{true\ negative}{true\ negative + false\ positive}$$

Proportion of individuals with RR-TB receiving a treatment regimen that matches the susceptibility of their *M. tuberculosis* strain to FLQ ($\boldsymbol{q}$):

$$\alpha \times \mu_{FLQ\text{-}R} + (1 - \mu_{FLQ\text{-}R})$$

Proportion of individuals with RR-TB unnecessarily receiving DLM instead of a FLQ ($\boldsymbol{c}$):

$$(1 - \beta) \times (1 - \mu_{FLQ\text{-}R})$$

S4 Bootstrap Validation

To conduct the internal validation of each model developed here, we followed the bootstrap validation procedure recommended by The TRIPOD Statement [5], as described below:

1. Develop the predictive model using the entire study population ($n = 540$) and calculate the apparent performance of interest (P), such as AUC-ROC, sensitivity, specificity, q, or c, as defined above. The apparent performance of a model refers to the performance of the model directly calculated using dataset that is used to train the model.
2. For $i = 1$ to 200:
   a. Create a bootstrap sample by sampling $n = 540$ individuals (from the original dataset) with replacement.
   b. Develop a bootstrap predictive model based on this bootstrap sample and calculate the apparent performance ($P_{i,sample}$), using the same feature selection and classification algorithms used in Step 1.
   c. Calculate the performance of the constructed bootstrap predictive model on the original sample ($P_{i,test}$).
   d. Calculated the optimism (O_i) by subtracting the test performance from the bootstrap performance: $O_i = P_{i,sample} - P_{i,test}$.
3. Calculate the average optimism: $\bar{O} = \sum_{i=1}^{200} O_i \,/200$.
4. Calculate the optimism-corrected performance as $P_{corrected} = P - \bar{O}$ with the $(1-\alpha)100\%$ bootstrap confidence interval of $[P - \delta_{\alpha/2}, P - \delta_{1-\alpha/2}]$, where $\delta_{\alpha/2}$ is the $(1-\alpha/2)100^{\text{th}}$ percentile of $\{O_1, O_2, \dots, O_{200}\}$.

S5 Cross Validation

Per RIPOD's recommendations, we use optimism-corrected performance measures (e.g., optimism-corrected AUC-ROC, optimism-corrected sensitivity, and optimism-corrected specificity) to evaluate the performance of different models (see §S4). Nonetheless, to evaluate if the estimates for AUC-ROC would change if a different validation algorithm were used, we also provide estimate of AUC-ROC calculated using 5-fold cross-validation (Table A). We, however, note that given the small size of our dataset and imbalance classes, we were not able to perform higher-fold cross validation to estimate AUC-ROC; hence, these estimates should be interpreted with caution.

Table A. The estimated area under the receiver operating characteristic curve (AUC-ROC) calculated using 5-fold cross-validation, for predictive models developed by different machine learning algorithms and feature selection methods.

**Machine learning model**	Logistic Regression			Neural Network	Random Forest	
**Feature selection method**	Recursive Feature Elimination	L_1 Regularization	Permutation Importance	Permutation Importance	Recursive Feature Elimination	Permutation Importance
**Model without information on local prevalence of resistance to FLQs**	0.61 (0.55, 0.70)	0.57 (0.49, 0.62)	0.59 (0.46, 0.78)	0.59 (0.56, 0.65)	0.54 (0.43, 0.67)	0.57 (0.48, 0.66)
**Model with information on local prevalence of resistance to FLQs**	0.68 (0.61, 0.74)	0.65 (0.59, 0.70)	0.65 (0.59, 0.72)	0.66 (0.59, 0.74)	0.63 (0.55, 0.69)	0.61 (0.44, 0.69)

S6 Impact on the selection of antibiotics

To evaluate whether the use of the predictive models developed here could improve the selection of antibiotics for patients with RR-TB, we estimated the net benefit of each model, which is defined as

$$U(\lambda, p) = \lambda q(p) - c(p),$$

where:

- p is the classification threshold ($p = 0$ is equivalent to sensitivity 0% and specificity 100%, and $p = 1$ is equivalent to sensitivity 100% and specificity 0%),
- $q(p)$ is the expected proportion of individuals with RR-TB who receive effective treatment (i.e., a regiment that is consistent with susceptibility of their *M. tuberculosis* strain to FLQs) if the classification threshold is set to p,
- $c(p)$ is the expected proportion of individuals with RR-TB who unnecessarily receive DLM (instead of an FLQ) if the classification threshold is set to p; and
- λ is a trade-off threshold that represent the policymaker's willingness to accept an increase in the proportion of individuals who unnecessarily receive DLM (i.e., $c(p)$) in order to increase the proportion of individuals who receive effective treatment (i.e., $q(p)$).

Following the WHO-recommended standardized regimen [6] corresponds to the scenario where all patients with RR-TB are assumed to be infected with a *M. tuberculosis* strain that is susceptible to FLQs. This strategy results in the net benefit of:

$$U_0(\lambda) = \lambda(1 - \mu_{FLQ\text{-}R}).$$

A predictive model with sensitivity $\alpha(p)$ and specificity $\beta(p)$ as the function of the classification threshold p results in the net benefit of:

$$U(\lambda, p) = \lambda q(p) - c(p)$$
$$= \lambda\left[\alpha(p) \times \mu_{FLQ\text{-}R} + \left(1 - \mu_{FLQ\text{-}R}\right)\right] - \left(1 - \beta(p)\right) \times \left(1 - \mu_{FLQ\text{-}R}\right).$$

To evaluate whether a predictive model would improve the selection of antibiotics for patients with RR-TB, we measured the utility of each model using the incremental net benefit, defined as:

$$\Delta U(\lambda, p) = U(\lambda, p) - U_0(\lambda)$$
$$= \lambda\left[\alpha(p) \times \mu_{FLQ\text{-}R}\right] - \left(1 - \beta(p)\right) \times \left(1 - \mu_{FLQ\text{-}R}\right).$$

For a given trade-off threshold λ, we chose a classification threshold p^* that maximizes $\Delta U(\lambda, p)$ as defined above. Figure 4 displays the optimism-corrected $\Delta U(\lambda, p^*(\lambda))$, using the bootstrap validation algorithm of §S4, for varying values of λ.

S7 Interpreting results of the random forest model with features identified by recursive feature elimination

The random forest model with features identified by recursive feature elimination has the second-best OC-AUC-ROC among all feature selection and classifier combinations. The top five most frequently selected features for the random forest model identified by recursive feature elimination include: age, number of household contacts 18 or younger, high district prevalence of FLQ-resistance, number of household contacts, and satisfactory living condition (Figure C). The top four features were consistent between random forest model with recursive feature elimination and neural network model with permutation importance.

Similar to the neural network model reported in the main manuscript, lowering the classification threshold of classifier increases the sensitivity of the model but decreases its specificity (Figure D, Panel A). Therefore, the tradeoff also exists between the proportion of patients with RR-TB who receive appropriate treatment regimen and the proportion of patients who may be unnecessarily treated with DLM (Figure D, Panel B). Depending on the policymaker's willingness to this tradeoff, Figure E displays the classification thresholds that maximizes the net benefit of random forest models for different values of trade-off threshold λ. The random forest model had statistically higher net benefit than the current strategy of using the standardized treatment regimen for all patients with RR-TB for trade-off thresholds $\lambda \geq 2.0$.

S8 Figures

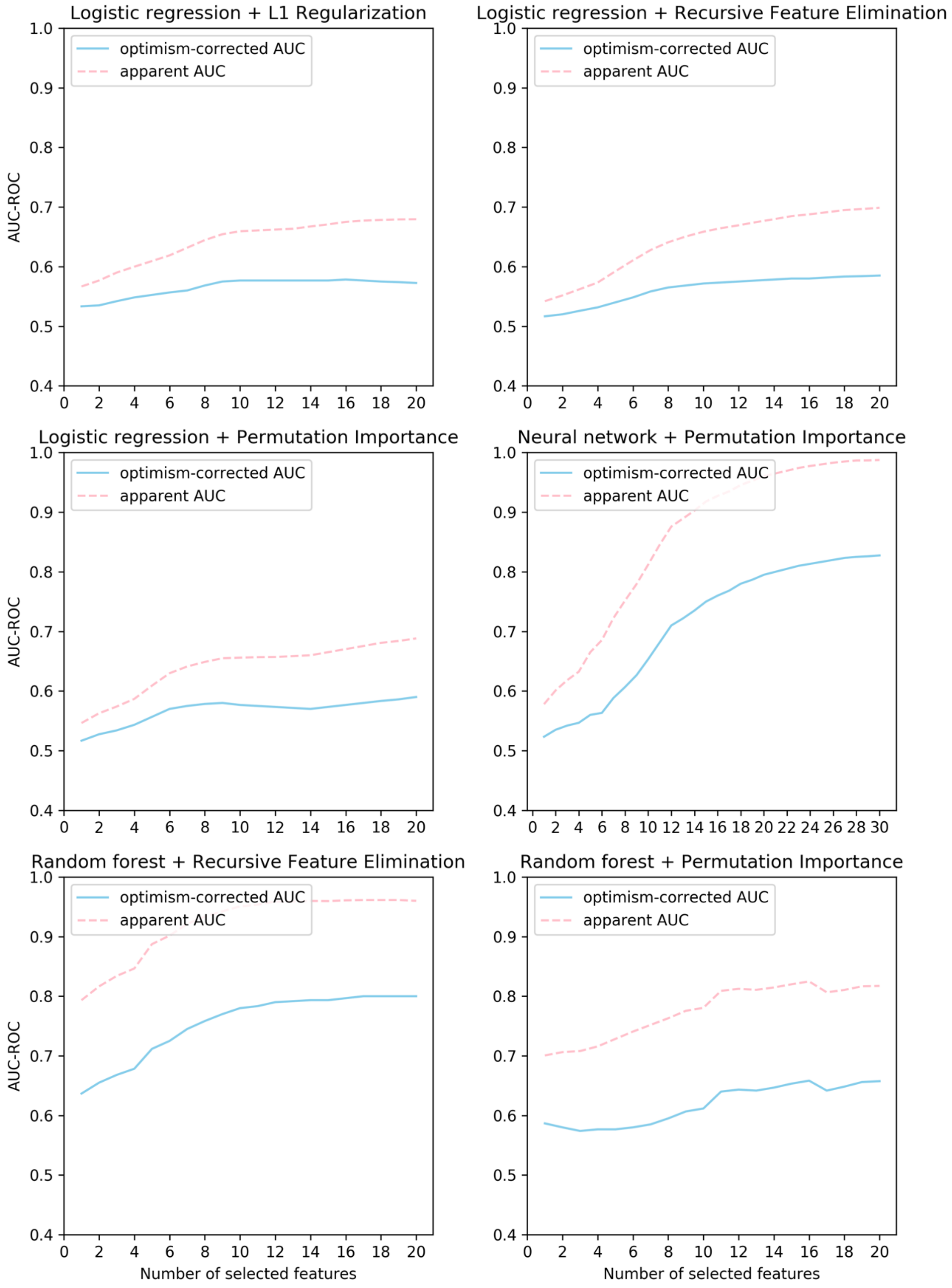

Figure A. The estimated area under the ROC curves (AUC-ROC) of predictive models that did not account for the local prevalence of resistance to FLQs, identified by different feature selection methods.

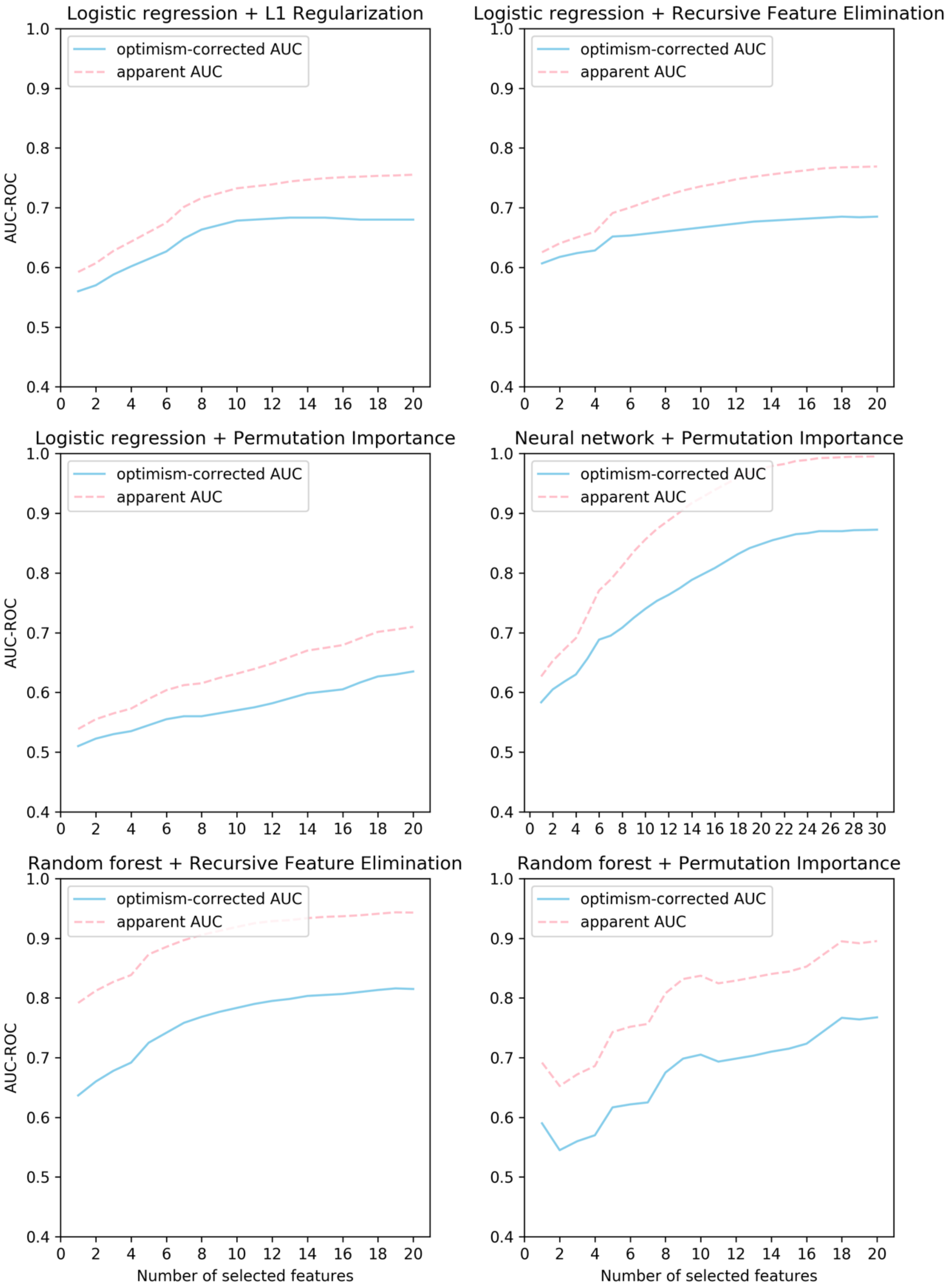

Figure B. The estimated area under the ROC curves (AUC-ROC) of predictive models that accounted for the local prevalence of resistance to FLQs, identified by different feature selection methods.

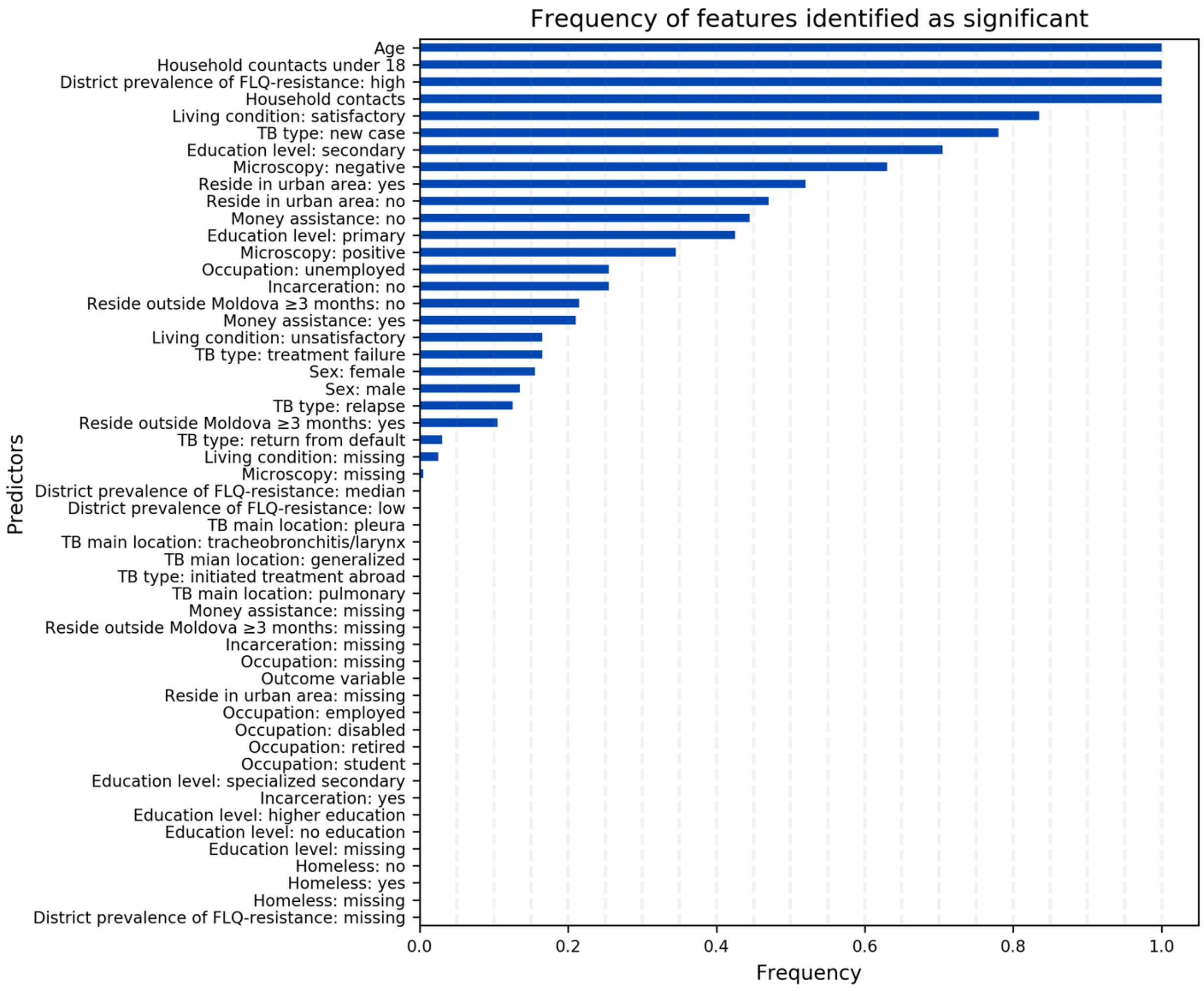

Figure C. The frequency of features identified as important using recursive feature elimination algorithm and random forest classifier among 200 bootstrap iterations (see §S4 in Supplement).

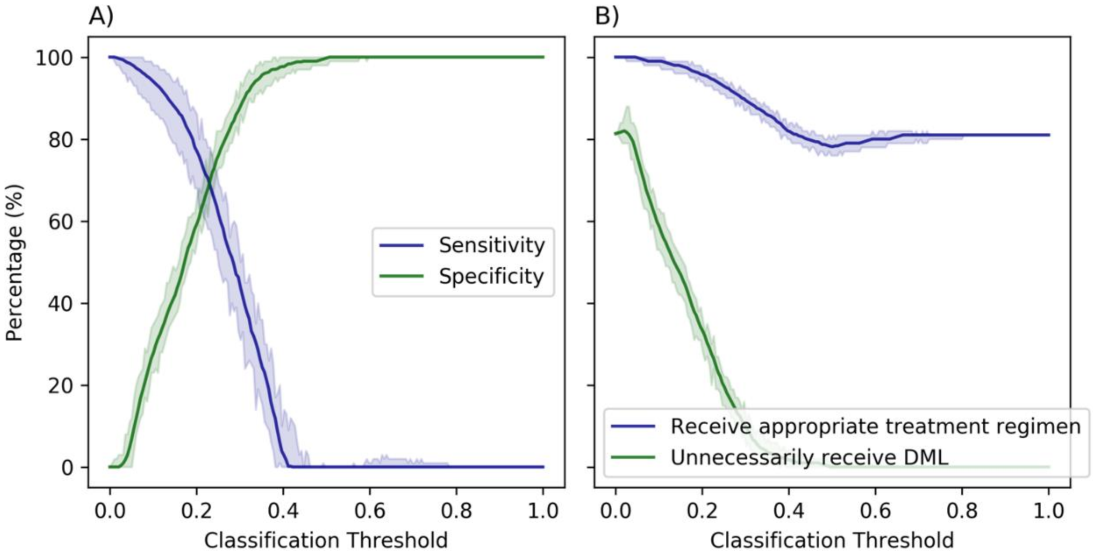

Figure D. Evaluating the performance of the random forest model that accounts for the local prevalence of resistance to FLQs using features identified by recursive feature elimination for varying classification threshold. The impact of the classification threshold on the optimism-corrected sensitivity and specificity is displayed in Panels A; the impact of the classification threshold on the optimism-corrected proportion of individuals receiving an appropriate treatment regimen (i.e., a regiment that is consistent with susceptibility of a patient's M. tuberculosis strain to FLQ) and on the optimism-corrected proportion of individuals who are unnecessarily treated with delamanid (DLM) is displayed in Panels B. The regions represent 95% bootstrap confidence intervals.

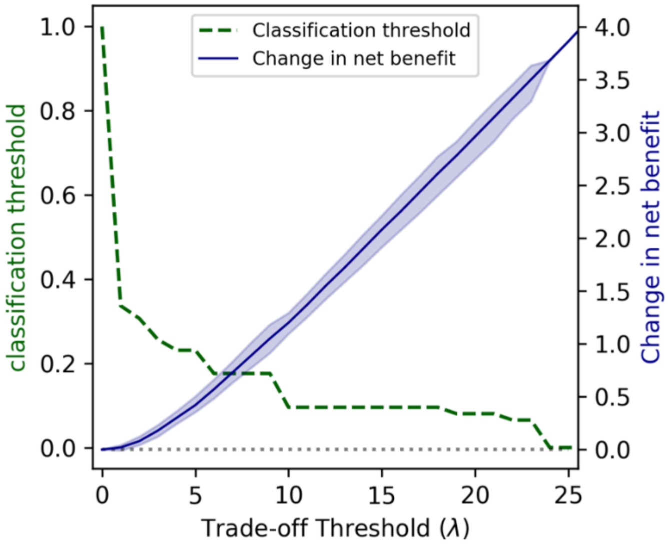

Figure E. The optimal choice of the classification threshold for varying values of the policymaker's trade-off threshold and the optimism-corrected utility of the random forest model to determine whether FLQs should be included or replaced with DLM for a patient with RR-TB. The model's utility is measured as the change in net benefit with respect to the strategy that uses the standardized treatment regimens for all patients with RR-TB. The trade-off threshold λ represents the percentage point increase in the proportion of individuals unnecessarily treated with DLM that the policymaker is willing to tolerate to increase the proportion of individuals who receive appropriate treatment by 1 percentage point. The regions represent 95% bootstrap confidence intervals.

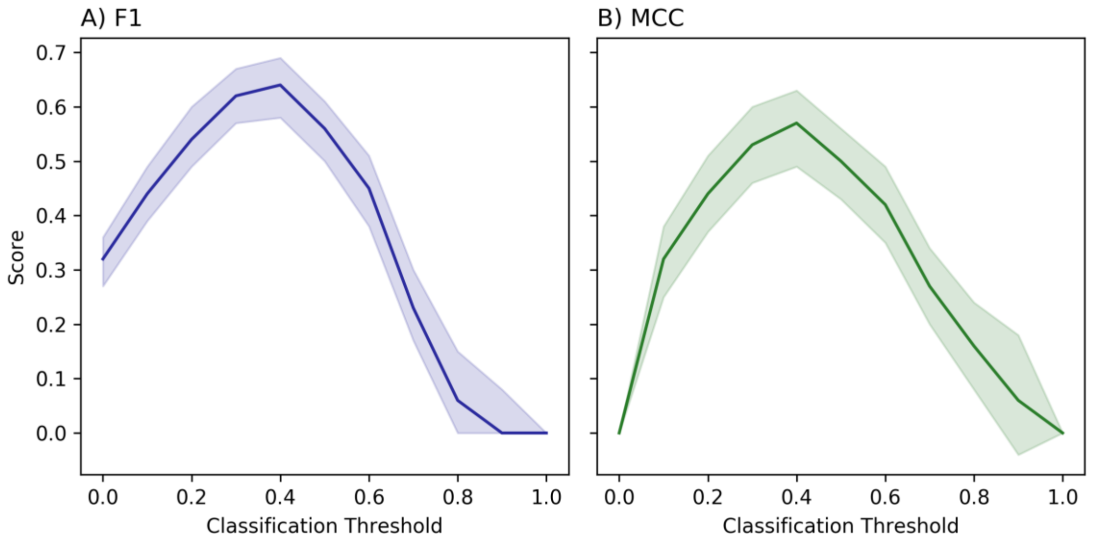

Figure F. The estimates F1 and Matthews correlation coefficient (MCC) scores for varying classification threshold for our final model (neural network classifier and permutation importance algorithm).

Reference

1. Pedregosa et al. Scikit-learn: Machine Learning in Python. JMLR 12, pp. 2825-2830, 2011.
2. Jović A, Brkić K, Bogunović N, editors. A review of feature selection methods with applications. 2015 38th international convention on information and communication technology, electronics and microelectronics (MIPRO); 2015: Ieee.
3. Vidaurre D, Bielza C, Larrañaga P. A survey of L1 regression. International Statistical Review. 2013;81(3):361-87.
4. Gregorutti B, Michel B, Saint-Pierre P. Correlation and variable importance in random forests. Statistics and Computing. 2017;27(3):659-78.
5. Moons KG, Altman DG, Reitsma JB, Ioannidis JP, Macaskill P, Steyerberg EW, et al. Transparent Reporting of a multivariable prediction model for Individual Prognosis or Diagnosis (TRIPOD): explanation and elaboration. Ann Intern Med. 2015;162(1):W1-73. Epub 2015/01/07. doi: 10.7326/m14-0698. PubMed PMID: 25560730.
6. World Health Organization. WHO consolidated guidelines on drug-resistant tuberculosis treatment. 2019.
