# Supplementary material for: Predicting resistance to fluoroquinolones among patients with rifampicin-resistant tuberculosis using machine learning methods
Source: PLOS Digit Health. 2022 Jun 30;1(6):e0000059. doi: 10.1371/journal.pdig.0000059 (PMC9518704; doi:10.1371/journal.pdig.0000059)
Supplement: Attachment — Submitted filename: Response Letter.pdf [file pdig.0000059.s004.pdf]

Yale SCHOOL OF PUBLIC HEALTH
Department of Health Policy and Management

Reza Yaesoubi, Ph.D.
Assistant Professor
Yale School of Public Health

courier
350 George Street, Room 308
New Haven, CT 06511

203 785-6982
reza.yaesoubi@yale.edu

March 3, 2022

Dear Professor Majumder:

Thank you for the opportunity to submit a revised version of our manuscript (tracking number: PDIG-D-21-00020): "Predicting resistance to fluoroquinolones among patients with rifampicin-resistant tuberculosis using machine learning methods."

We would like to thank reviewers for their constructive comments, which have contributed substantially to improving the content and the presentation of our manuscript. We have addressed each of the suggestions and concerns, and we have provided point-by-point responses in the attached response letter.

Thank you again for your consideration of this revised manuscript. We look forward to your evaluation and are available if questions arise.

Sincerely,

Reza Yaesoubi, on behalf of co-authors.

INNOVATION THROUGH COLLABORATION
100
Yale School of Public Health
1915–2015

Responses to Reviewer 1

"This work used single site RR-MTB data to build machine learning models. The input includes heterogeneous features collected for national surveillance, e.g. demographics, TB testing, etc. A trade-off strategy is proposed for determining classification threshold. Several comments are as follows."

(1) "P6, the equation for determining classification threshold. If FLQ-resistance is positive, the q(p) denotes true negative (FLQ-susceptible case that receives the treatment regime with FLQ properly) and c(p) is false positive (FLQ-susceptible case that receives DLM unnecessarily). Contrarily, it is stated in P8 that the threshold is determined through trade-off between false positive and false negative. Please clarify"

Response: Thank you for identifying this error. We have corrected the sentence in the discussion section:

> "The optimal choice of this threshold requires a trade-off between the costs of <u>a true negative and a false positive</u> diagnosis."

(2) "The most important details of experiments are missing, e.g., data split, the parameter setting of different machine learning models and feature selection scheme. Therefore, it's difficult to answer the following questions: (1) which model is the best? (2) how are the reported results obtained?"

Response: Thank you for raising this concern. We made several changes, as described below, to provide additional details throughout the manuscript. As noted in the manuscript, we followed the guidelines for the Transparent Reporting of a multivariable prediction model for Individual Prognosis Or Diagnosis (TRIPOD) to develop and evaluate the predictive models described in our paper. Per TRIPOD's recommendation, we used the bootstrap validation procedure (described in §S4 of the Supplement) to estimate the optimism-corrected area under the receiver operating characteristic curve (OC-AUC-ROC), which we used to identify the model with the best performance. We describe in section "Model development and evaluation" that we used this measure to select the model with the best performance.

> "We compared the performances of models identified through different feature selection methods based on the estimated OC-AUC-ROC and selected the model with the highest estimated OC-AUC-ROC as the final model."

We note that the bootstrap validation algorithm recommended by TRIPOD does not require splitting the dataset (as explained in §S4 of the Supplement). We now discuss this at a greater length under "Model development and evaluation":

> "To assess the internal validity of our models, we followed the bootstrap validation procedure recommended by The TRIPOD Statement (see §S4) to estimate the optimism-corrected area under the receiver operating characteristic curve (OC-AUC-ROC), and the optimism-corrected sensitivity, specificity, F1 score, and Matthews correlation coefficient (MCC). <u>Compared to randomly splitting the dataset into model development and model validation sets, the bootstrap validation approach recommended by the TRIPOD Statement is shown to be a stronger approach as it utilizes the entire dataset for model development and validation.</u> Given that the relatively small size of our dataset (N=540) would not allow for conducting temporal validation, we use the bootstrap validation approach recommended by the TRIPOD Statement to obtain estimates the performance measures listed above."

We also revised the section "S2 Machine learning and feature selection algorithms" in the Supplement to provide additional details about parameter settings of different machine learning methods and feature selection algorithms:

> "We used the scikit-learn package to train logistic regression, neural network, and random forest models.[1] We set the class weight of the logistic regression models to be "balanced". Neural network models were trained using the lbfgs solver with tanh activation functions and one hidden layer containing as many nodes as the number of features + 2. For the random forest models, we set the number of trees to be 100 and the minimal number of samples required to be at a leaf node to be 5.
>
> To identify features with important predictive power and to remove features that would diminish the model's accuracy, we used recursive feature elimination (RFE),[2] L_1 regularization (L1),[3] and permutation importance (PI)[4] as feature selection methods for different classifiers. We used RFE, L1 and PI for logistic regression models, RFE and PI for random forest models, and PI for neural network model.[1] For L1, we set the regularization parameter to 0.2 and for PI, we set number of times a feature is randomly shuffled to 10.
>
> To determine the optimal number of features using RFE and PI, we checked how the optimism-corrected estimates of AUC-ROC (calculated using the algorithm described below in §S4) varied by the number of features included (Figure S1 and Figure S2). We chose the smallest number of features after which the optimism-corrected estimate of AUC-ROC became stable. To characterize the importance of each feature, we e recorded the number of times each feature was identified as important within the iterations of the bootstrap algorithm describe below."

(3) "What are the selected features that generate the best performance?"

Response: We now provide the list of features included in our final model and a new figure (figure 2) to display the frequency of features identified as important through the iterations of the bootstrap validation algorithm (described in §S4 of the Supplement). This figure provides insights on the relative importance of different features. The following text and figure are added to the Results section:

> "Our final model included the following features: age, number of household contacts, number of household contacts 18 or younger, living in district with low, medium, or high prevalence of FLQ-resistance, TB type (new or relapse), education level (secondary or primary), if unemployed, results of microscopy test, whether residing in an urban area, and whether the living condition is satisfactory. These features were selected as important in more than 50% of bootstrap iterations used to estimate OC-AUC-ROC of this model, as described in §S4 of the Supplement (Figure 2)."

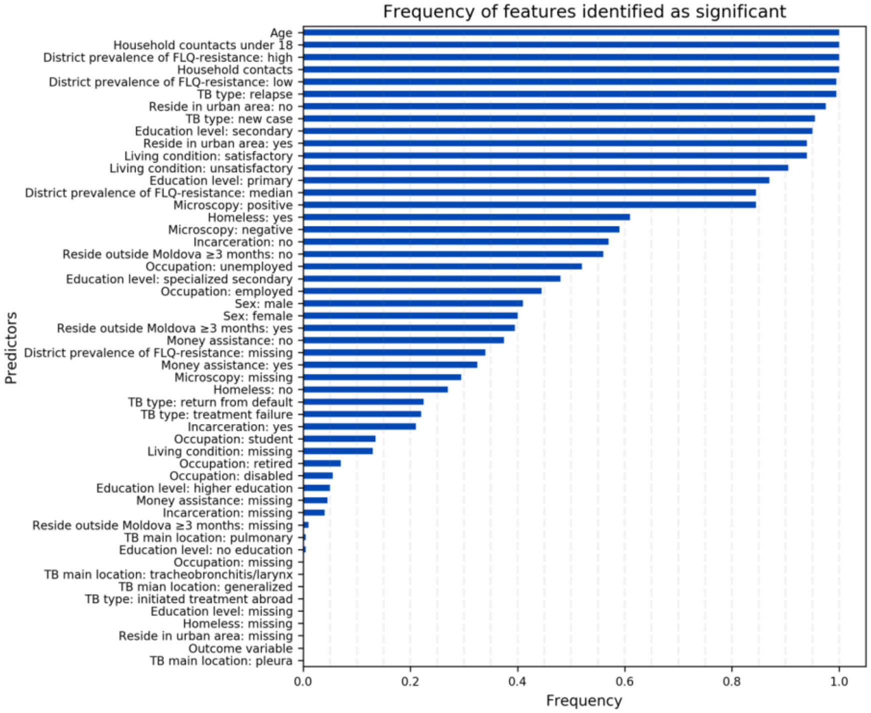

Figure 2. The frequency of features identified as important through iterations of the bootstrap validation algorithm (§S4 in Supplement) to evaluate the OC-AUC-ROC of our final model (neural network classifier and permutation importance algorithm).

(4) "The most important pre-processing details are missing. How are the categorical input features converted to numerical input?"

<u>Response</u>: We provide additional details related to pre-processing steps in the section "S1.2 Preprocessing" of the Supplement.

> "We used <u>one-hot encoding to incorporate nominal categorical features (e.g., occupation, education, TB type)</u>. We standardized the only continuous feature in our dataset (i.e., age). For feature 'number of household contacts' we grouped entities with values ≥ 11 into a single stratum and treated these features as <u>ordinal categorical predictors (i.e., replacing its values with 0, 1, 2, …</u>). For feature 'number of household contacts 18 or younger', we grouped entities with values ≥ 8 into a single stratum. We extrapolated the missing entries for 'number of household contacts' and 'number of household contacts 18 or younger' by replacing them with the mean values of each column. <u>For the feature 'residing in a district with low, median, or high prevalence of resistance to FLQs' we replaced low, medium, and high values with 0, 1, 2.</u>"

Responses to Reviewer 2

"We have read "Predicting resistance to fluoroquinolones among patients with rifampicin-resistant tuberculosis using machine learning methods" by You et al. The authors developed a machine-learning predictor for fluoroquinolone resistance of rifampicin resistance tuberculosis (TB) based on patient demographics, TB clinical information and disease prevalence features. Predictions from this model could be used to select antibiotics more rationally for treatment of TB in the future. We appreciated the author's clear description of the methods, results and implications as well as their honest discussion of limitations. Overall, we only have minor questions regarding feature interpretation, model training and the necessity of machine learning for this application given the relative few number of features."

(1) "We were excited to see that the models were trained using demographic and surveillance information (rather than genomic data) because it could reasonably be rolled out quickly without new equipment. However, we were disappointed that relatively little time was spent discussing the most important features to model predictions. We feel the manuscript would benefit from more interpretation of important features leading to model predictions (e.g. was homelessness a major factor? What about TB prevalence? We are familiar with some nice ways to evaluate feature importance for random forests, e.g. permutation, MDA, etc. A description of feature importance for the best models would be a valuable addition to this manuscript."

<u>**Response**</u>: Thank you for raising this important point. We have included additional information about the set of features that our final model includes and a new figure (Figure 2, also included above in our response to Reviewer 1's third comment) to display the relative importance of different features considered in our analysis: The following text is added to Result section:

> "Our final model included the following features: age, number of household contacts, number of household contacts 18 or younger, living in district with low, medium, or high prevalence of FLQ-resistance, TB type (new or relapse), education level (secondary or primary), if unemployed, results of microscopy test, whether residing in an urban area, and whether the living condition is satisfactory. These features were selected as important in more than 80% of bootstrap iterations used to estimate OC-AUC-ROC of this model, as described in §S4 of the Supplement (Figure 2)."

(2) "The authors don't discuss model cross validation in-depth but based on the methods section it looks like they did some cross validation with TRIPOD recommended bootstrapping. Have the authors compared this method with others like leave-one-out or KFolds? We understand that the sample size is relatively smally and class sizes are unbalanced but it would still be nice to understand how model predictions fare with a larger hold-out group. This would also inform how potentially generalizable such a model might be to other regions."

<u>**Response**</u>: Thank you for making this suggestion. Per TRIPOD's recommendations, we presented our results based on optimism-corrected performance measures (e.g., optimism-corrected AUC-ROC, optimism-corrected sensitivity, and optimism-corrected specificity). In the revised manuscript, we now provide the results of 5-fold cross validation for all models considered here. We note that test AUC-ROC cannot not be calculated for the leave-one-out design and that given the small size of our dataset and the imbalanced classes, we were not able to perform higher-fold cross validation to estimate AUC-ROC. Therefore, these

estimates should be interpreted with caution. The results of cross validation are provided in section "S5 Cross Validation" of the Supplement and in Table S1 included below.

Table S1. The estimated area under the receiver operating characteristic curve (AUC-ROC) calculated using 5-fold cross-validation, for predictive models developed by different machine learning algorithms and feature selection methods.

**Machine learning model**	Logistic Regression			Neural Network	Random Forest	
**Feature selection method**	Recursive Feature Elimination	L_1 Regularization	Permutation Importance	Permutation Importance	Recursive Feature Elimination	Permutation Importance
**Model without information on local prevalence of resistance to FLQs**	0.61 (0.55, 0.70)	0.57 (0.49, 0.62)	0.59 (0.46, 0.78)	0.59 (0.56, 0.65)	0.54 (0.43, 0.67)	0.57 (0.48, 0.66)
**Model with information on local prevalence of resistance to FLQs**	0.68 (0.61, 0.74)	0.65 (0.59, 0.70)	0.65 (0.59, 0.72)	0.66 (0.59, 0.74)	0.63 (0.55, 0.69)	0.61 (0.44, 0.69)

We have also added the following text to the Results to explain the rationale for using the bootstrap validation method recommended by the TRIPOD's statement:

> "Compared to randomly splitting the dataset into model development and model validation sets, the bootstrap validation approach recommended by the TRIPOD Statement is shown to be a stronger approach as it utilizes the entire dataset for model development and validation. Given that the relatively small size of our dataset ($N = 540$) would not allow for conducting temporal validation, we use the bootstrap validation approach recommended by the TRIPOD Statement to obtain estimates the performance measures listed above."

(3) "The authors don't discuss the results from their neural networks much. We are interested in their opinion of why the neural networks did not perform as well as other methods. Why did they opt to remove prevalence of FLQ from this model in particular?"

Response: Thank for raising this concern, which prompted us to explore ways to optimize the performance of neural network models. We found that using hyperbolic tangent (instead of logistic) activation function would substantially improve the performance of neural network models in our study. Since neural network models turned out to perform better than random forest models, we updated our results and conclusions based on these new results. We finally note that we evaluated the performance of all models once with and once without the feature representing the local prevalence of FLQ-resistance (Table 2).

Responses to Reviewer 3

"The manuscript: "Predicting resistance to fluoroquinolones among patients with rifampicin-resistant tuberculosis using machine learning methods" describes an effort to detect resistance to second line fluoroquinolones by using data from rifampicin-resistant TB samples as delineated by GeneXpert-MTB/RIF, in samples present in Moldova. The authors use the optimism corrected AUROC metric to justify the performance of their models, which were built using logistic regression, neural network and random forest algorithms. Below are my general comments per section."

(1) "**Methods: Data source and study population**. It is unclear in the Methods section as to why and how there are cases in Table one which have been diagnosed with RR-TB through Xpert-MTB/RIF, but were negative for rifampicin resistance, presumably using other DST methods."

Response: Thank you for raising this issue. To clarify the definition of "Rifampicin resistance" in Table 1, we renamed this item to "Rifampicin resistance detected by culture" and added the following text to the footnote of Table 1 to emphasize that resistance to rifampicin was assumed if either or both LJ and MGIT culture tests were positive. We also note that given that these culture tests are not perfect, it is possible that a small number of individuals who are diagnosed with rifampicin-resistant TB through Xpert-MTB/RIF have a negative culture test.

> "Resistance to rifampicin was assumed if either or both LJ and MGIT culture tests were positive (see S1.1 for additional details). We note that given that these culture tests have imperfect sensitivity and specificity, it is possible that a small number of individuals who are diagnosed with rifampicin-resistant TB through Xpert-MTB/RIF have a negative culture test."

(2) "**Methods: Predictors and outcomes**. Overall, the idea behind using Expert-MTB/RIF data to detect resistance to FLQs is innovative, however, I am concerned about the general interpretability of the model considering the range of features used – particularly as certain features may be correlated to disease prevalence, but not necessarily contribute to FLQ resistance. Further to that, it is unclear how the authors account for missing data in cases where only partial demographic, TB-related or microbiological tests are present. Supplementary section 1.2 briefly brushes on this point – however the authors do not explicitly refer to this in the main text, while it is unclear whether supervised or semi-supervised machine learning was adopted to develop the models, considering missing data."

Response: To improve the interpretability of our final model, we have included a new figure (Figure 2, also included above in our response to Reviewer 1's third comment) to display the relative importance of different features considered in our analysis: The following text is also added to Result section:

> "Our final model included the following features: age, number of household contacts, number of household contacts 18 or younger, living in district with low, medium, or high prevalence of FLQ-resistance, TB type (new or relapse), education level (secondary or primary), if unemployed, results of microscopy test, whether residing in an urban area, and whether the living condition is satisfactory. These features were selected as important in more than 50% of bootstrap iterations used to estimate OC-AUC-ROC of this model (Figure 2)."

We revised the section "Predictors and outcomes" to provide more information about how missing data were handled:

> "Table 1 displays the distribution of values that these features take among patients with RR-TB, and patients with RR- and FLQ-resistant TB. To prepare our dataset, we coded entities with no or unrealistic values as "missing." We note that features age, sex, and TB type did not have a missing value (Table 1). When training and evaluating our predictive models, we extrapolated the missing entries for feature 'number of household contacts' and 'number of household contacts 18 or younger' by replacing them with the mean values of each column. For all categorical features, as the occurrence of "missing" values may not be completely random, we kept all records with a "missing" value for these features when training and evaluating our predictive models."

Finally, under the section "Model development and evaluation," we now explicitly state that we used supervised machine learning methods in our analysis:

> "We considered three supervised machine learning models: logistic regression, neural network, and random forest."

(3) "**Methods: Model development and evaluation**. The readers would benefit from an explanation of the differences between the three feature selection methods adopted. Further to that, it would also be useful for the readers to understand why the TRIPOD recommendations were used, rather than separating the data into train/test and using metrics other than OC-AUROC to assess model performance. This is especially because training and testing on the same data offers no generalizability potential for the model (as the readers highlight), meaning that its real-world clinical utility cannot be quantified. It would perhaps be more useful if both types of models were presented, and final features also compared. Additionally, reporting only OC-AUROC for the TRIPOD-based model is not enough to assess performance, and more balanced features, like MCC or F1 score may prove additional information."

Response: Thank you for these suggestions. To explain the differences between the three feature selection methods considered in our analysis, we added the following text to section "S2 Machine learning and feature selection algorithms" in the Supplement:

> "The L_1 regularization imposes a penalty on the sum of absolute values of feature coefficients and can only be applied to logistic regression models. It seeks to lower the coefficient of unimportant features to zero and features with non-zero coefficients are identified as important features.[31] The recursive feature elimination (RFE) selects features by dropping the least important feature recursively until the specified number of features are selected. It ranks features based on weights assigned to features (e.g., the coefficients of a regression model or the feature importance of a random forest model).[1] It is shown to perform well for random forest models in the presence of correlated features.[2] Since it is a greedy algorithm, once a variable is excluded through the process, it will not be added back. Furthermore, RFE is not applicable to neural network models. The permutation importance (PI) ranks feature based on the improvement in the model score when a single feature value is randomly shuffled.[1] PI can be used for any classification method when the data is tabular.[1] Therefore, we were able to apply this feature selection algorithm to three classifiers considered in our study."

To provide more details about why we used the TIRPOD recommended approach to evaluate the interval validity of our predictive models, we added the following text to section "Model development and evaluation"

> "To assess the internal validity of our models, we followed the bootstrap validation procedure recommended by The TRIPOD Statement (see §S4) to estimate the optimism-corrected area under the receiver operating characteristic curve (OC-AUC-ROC), and the optimism-corrected sensitivity, specificity, F1 score, and Matthews correlation coefficient (MCC).[46] Compared to randomly splitting the dataset into model development and model validation sets, the bootstrap validation approach recommended by the TRIPOD Statement is shown to be a stronger approach as it utilizes the entire dataset for model development and validation. Given that the relatively small size of our dataset ($N = 540$) would not allow for conducting temporal validation, we use the bootstrap validation approach recommended by the TRIPOD Statement to obtain estimates the performance measures listed above."

In the revised manuscript, we now provide estimates of AUC-ROC for all models considered here using a 5-fold cross validation approach (in addition to the optimism-corrected estimates, which we have provided before using the TRIPOD's recommended approach). The details are provided in a new section in the Supplement (S5 Cross Validation) and the results are shown in Table S1 (also included above in our response to Reviewer 2's second comment).

We also provide a new figure displaying the values of MCC and F1 score for our final model for varying classification thresholds (Figure S6 in the Supplement and below).

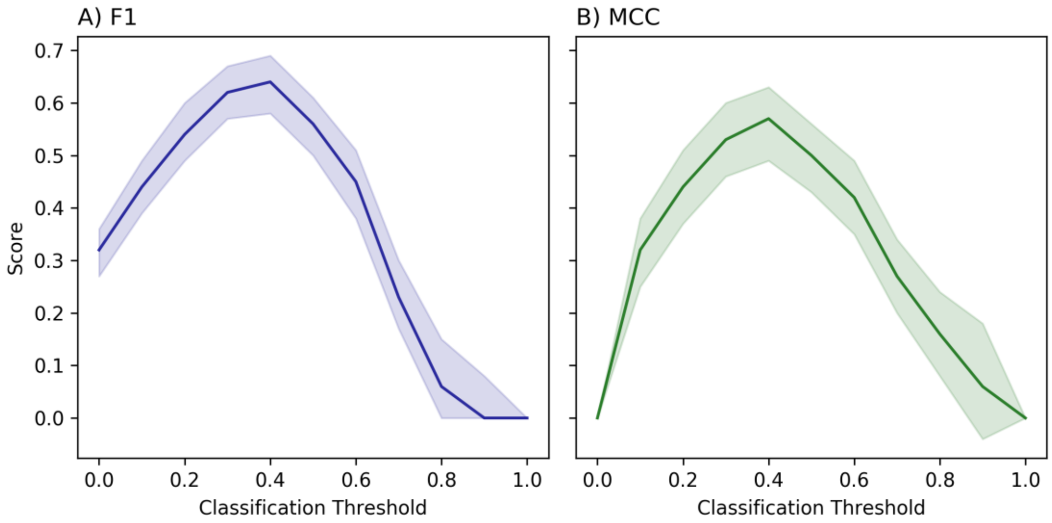

Figure S6. The estimates F1 and Matthews correlation coefficient (MCC) scores for varying classification threshold for our final model.

(4) "**Results**. While a list of selected features has been made available by the authors in the supplementary files, and highlighted in the discussion, the authors do not investigate the importance and relevance of these features further, which would be especially informative to interpret the model itself, and its potential utility in the clinic. It is not especially clear how the authors investigated the replacement of FLQs with DLM – was it through mathematical modelling or machine learning? Also, is there a possible reason why resistance to

one FLQ does not warrant switching to another FLQ before using the last-line drug DLM? This needs to be made clear within the main text."

Response: Thank you for raising these important points. We have included additional information about the set of features that our final model includes and a new figure (Figure 2, also included above in our response to Reviewer 1's third comment) to display the relative importance of different features considered in our analysis: The following text is added to Result section:

> "Our final model included the following features: age, number of household contacts, number of household contacts 18 or younger, living in district with low, medium, or high prevalence of FLQ-resistance, TB type (new or relapse), education level (secondary or primary), if unemployed, results of microscopy test, whether residing in an urban area, and whether the living condition is satisfactory. These features were selected as important in more than 50% of bootstrap iterations used to estimate OC-AUC-ROC of this model (Figure 2)."

Regarding the decision to replace FLQs with DLM when resistance to FLQs is suspected, we followed the hierarchy of the WHO grouping of anti-TB agents, noting that it is not recommended to replace a FLQ with another FLQ if resistance to any FLQs is detected.[5] Furthermore, in the first paragraph under "Impact on selection of antituberculous medications" we provided a more detailed definition of an "appropriate treatment regimen" in the context of our analysis:

> "Following the hierarchy of the WHO grouping of anti-TB agents,9 we assume that when resistance to FLQs is suspected, FLQs is replaced with delamanid (DLM). The use of predictive models to decide whether FLQs should be included or replaced with DLM, could improve the probability that a patient with RR-TB receives an appropriate treatment regimen (i.e., a treatment regimen that includes FLQs when susceptible to FLQs and that includes DLM in place of FLQs when resistance to FLQs). However, predictive models with low specificity would increase the unnecessary use of DLM, which consequently raises the risk for the emergence of additional resistance."

(5) "**Discussion**. While suggesting the replacement of FLQs with DLM and the different thresholds applied, it is important for the readers to understand the implications of using DLM haphazardly in the clinic – meaning that stats pertaining to current TB resistance (if any) to DLM need to be highlighted, as well as the importance of DLM stewardship to increase its clinical longevity. This includes a discussion on what a >= 2.0 percent point increase would mean if policymakers chose to take the risk."

Response: Thank you for raising this important point. We added the following text to the Discussion section to further highlight the tradeoff between using DML to improve the treatment outcomes of patients with RR-TB while also ensuring its clinical longevity.

> "We measured the utility of a predictive model based its impact on the proportion of patients with RR-TB who would receive an appropriate treatment regimen or would be unnecessarily treated with DLM. The utility of predictive models should be ideally investigated using a cost-effectiveness analysis that properly quantifies the cost and health consequences of replacing FLQs with DLM, which could reduce the clinical longevity of DLM but improve the treatment outcomes of patients with RR-TB. Accounting for this tradeoff allows the decisionmaker to identify the optimal classification threshold based on their

tolerance in accepting a shorter clinical longevity for DLM if that leads to improving the treatment outcomes of patients with RR-TB."
