# Supplementary material for: Predicting resistance to fluoroquinolones among patients with rifampicin-resistant tuberculosis using machine learning methods
Source: PLOS Digit Health. 2022 Jun 30;1(6):e0000059. doi: 10.1371/journal.pdig.0000059 (PMC9518704; doi:10.1371/journal.pdig.0000059)
Supplement: Attachment — Submitted filename: PDIG-D-21-00020_reviewer_comments.docx [file pdig.0000059.s003.docx]

The manuscript: “Predicting resistance to fluoroquinolones among patients with rifampicin-resistant tuberculosis using machine learning methods” describes an effort to detect resistance to second line fluoroquinolones by using data from rifampicin-resistant TB samples as delineated by GeneXpert-MTB/RIF, in samples present in Moldova. The authors use the optimism corrected AUROC metric to justify the performance of their models, which were built using logistic regression, neural network and random forest algorithms.

Below are my general comments per section.

Methods: Data source and study population

It is unclear in the Methods section as to why and how there are cases in Table one which have been diagnosed with RR-TB through Xpert-MTB/RIF, but were negative for rifampicin resistance, presumably using other DST methods.

Methods: Predictors and outcomes

Overall, the idea behind using Expert-MTB/RIF data to detect resistance to FLQs is innovative, however, I am concerned about the general interpretability of the model considering the range of features used – particularly as certain features may be correlated to disease prevalence, but not necessarily contribute to FLQ resistance. Further to that, it is unclear how the authors account for missing data in cases where only partial demographic, TB-related or microbiological tests are present. Supplementary section 1.2 briefly brushes on this point – however the authors do not explicitly refer to this in the main text, while it is unclear whether supervised or semi-supervised machine learning was adopted to develop the models, considering missing data.

Methods: Model development and evaluation

The readers would benefit from an explanation of the differences between the three feature selection methods adopted. Further to that, it would also be useful for the readers to understand why the TRIPOD recommendations were used, rather than separating the data into train/test and using metrics other than OC-AUROC to assess model performance. This is especially because training and testing on the same data offers no generalizability potential for the model (as the readers highlight), meaning that its real-world clinical utility cannot be quantified. It would perhaps be more useful if both types of models were presented, and final features also compared. Additionally, reporting only OC-AUROC for the TRIPOD-based model is not enough to assess performance, and more balanced features, like MCC or F1 score may prove additional information.

Results

While a list of selected features has been made available by the authors in the supplementary files, and highlighted in the discussion, the authors do not investigate the importance and relevance of these features further, which would be especially informative to interpret the model itself, and its potential utility in the clinic.

It is not especially clear how the authors investigated the replacement of FLQs with DLM – was it through mathematical modelling or machine learning? Also, is there a possible reason why resistance to one FLQ does not warrant switching to another FLQ before using the last-line drug DLM? This needs to be made clear within the main text.

Discussion

While suggesting the replacement of FLQs with DLM and the different thresholds applied, it is important for the readers to understand the implications of using DLM haphazardly in the clinic – meaning that stats pertaining to current TB resistance (if any) to DLM need to be highlighted, as well as the importance of DLM stewardship to increase its clinical longevity. This includes a discussion on what a >= 2.0 percent point increase would mean if policymakers chose to take the risk.
